# Differential down-regulation of HLA-DR on monocyte subpopulations during systemic inflammation

**DOI:** 10.1186/cc8959

**Published:** 2010-04-13

**Authors:** Oh Yoen Kim, Antoine Monsel, Michèle Bertrand, Pierre Coriat, Jean-Marc Cavaillon, Minou Adib-Conquy

**Affiliations:** 1 Unit "Cytokines & Inflammation", Institut Pasteur, 28 rue Dr. Roux, Paris, 75015 France; 2 Department of Anesthesiology and Critical Care, Université Pierre et Marie Curie - Paris 6, and Centre Hospitalier Universitaire Pitié-Salpêtrière, Assistance-Publique, Hôpitaux de Paris, 47 bd de l'Hôpital, Paris, 75013 France

## Abstract

**Introduction:**

Decreased expression of human leukocyte antigen class II (HLA-DR) on monocytes is a hallmark of altered immune status in patients with a systemic inflammatory response syndrome (SIRS). So far, the analyses were mainly performed without taking into account monocytes subpopulations.

**Methods:**

We studied this modification on CD14^HIGH ^and CD14^LOW ^monocytes of 20 SIRS patients undergoing abdominal aortic surgery (AAS), 20 patients undergoing carotid artery surgery (CAS), and 9 healthy controls, and we investigated mediators and intracellular molecules that may be involved in this process.

**Results:**

HLA-DR on CD14^HIGH ^monocytes started to decrease during surgery, after blood reperfusion, and was further reduced post-surgery. In contrast, HLA-DR expression on CD14^LOW ^cells only decreased after surgery, and to a lesser extent than on CD14^HIGH ^monocytes. Negative correlations were found between the reduction of HLA-DR expression and the change in cortisol levels for both subpopulations, whereas a negative correlation between interleukin-10 (IL-10) levels and HLA-DR modulation was only observed for CD14^HIGH ^cells. In accordance with these *ex vivo *results, HLA-DR on CD14^HIGH ^and CD14^LOW ^monocytes of healthy donors was reduced following incubation with hydrocortisone, whereas IL-10 only acted on CD14^HIGH ^subpopulation. Furthermore, flow cytometry revealed that the expression of IL-10 receptor was higher on CD14^HIGH ^versus CD14^LOW ^monocytes. In addition, hydrocortisone, and to a lesser extent IL-10, reversed the up-regulation of HLA-DR induced by bacterial products. Finally, membrane-associated RING-CH-1 protein (MARCH1) mRNA, a negative regulator of MHC class II, was up-regulated in monocytes of AAS patients on Day 1 post-surgery, and in those of healthy subjects exposed to hydrocortisone.

**Conclusions:**

This study reveals that HLA-DR expression is modulated differently on CD14^HIGH ^(*classical*) versus CD14^LOW ^(*inflammatory*) monocytes after systemic inflammation.

## Introduction

Patients with non-infectious systemic inflammatory response syndrome (SIRS) or sepsis display an altered immune status, often referred to as *compensatory anti-inflammatory response syndrome *or CARS [1,2]. CARS is characterized by reduced *in vitro *lymphocyte proliferation [3], reduced *ex vivo *cytokine production upon activation of monocytes and neutrophils by endotoxin (lipopolysaccharide, LPS) [4,5], reduced Natural Killer (NK) cell activity [6], enhanced apoptosis of lymphocytes and dendritic cells [7], and profound modification of different cell surface markers. Among cell surface changes, the diminished expression of human leukocyte antigen class II (HLA-DR) on circulating CD14+ monocytes is a hallmark of altered immune status in patients after stressful insult (for example, trauma, severe surgery, hemorrhagic shock, pancreatitis, burn, and sepsis). Hershman et al. [8] showed in trauma patients that the decreased expression of HLA-DR was long-lasting and more pronounced in patients who developed sepsis, and dramatically more severe in those who ultimately died. While the levels of HLA-DR could not discriminate between survivors and non-survivors at diagnosis of sepsis, a few days later these levels were significantly lower in patients who died [9]. HLA-DR was also shown to be associated with the outcome in community acquired severe infections [10], patients with pancreatitis [11], patients with ruptured abdominal aortic aneurysm [12], and patients after cardiac surgery [13]. The most promising use of HLA-DR expression as a marker on CD14+ cells is its association with infection after non-infectious insults such as surgery [14], liver transplantation [15], trauma [16], pancreatitis [17], or burn injury [18]. In association with measurements of interleukin-10 (IL-10) in the plasma, HLA-DR levels can predict outcomes after nosocomial infections [16,19]. As stated by Fumeaux and Pugin [20], HLA-DR expression appears to be a robust marker of immune dysfunction in critically ill patients.

Among the mediators produced during inflammation, cortisol [21] and IL-10 [22] were shown to contribute to the down-regulation of HLA-DR on CD14+ cells. In parallel, IL-10 was shown to up-regulate the membrane-associated RING-CH-1 protein (MARCH1) [23], an ubiquitin E3 ligase that promotes the ubiquination and internalization of the HLA-DR β-chain, thus playing a major role in HLA-DR trafficking [24,25].

Different subsets of circulating monocytes have been described depending on the presence or absence of CD16 [26,27], and CX_3_CR1 [28], or the levels of CD14 expression [27,29,30]. CD14^LOW ^(CD16+) monocytes represent a minor subset in healthy donors, but their percentage substantially increases during sepsis [29]. So far, the analysis of HLA-DR has been rarely performed taking into account these different subpopulations. We therefore decided to investigate the modification of HLA-DR expression on CD14^HIGH ^and CD14^LOW ^cells of patients undergoing severe surgery. The analysis was performed at different timings during surgery and on the following days. Because HLA-DR appeared to be differently regulated on monocyte subpopulations, we also performed *in vitro *experiments to further identify mediators and intracellular molecules possibly involved in this process.

## Materials and methods

### Subjects and operation

Patients scheduled for abdominal aortic surgery (AAS) and carotid artery surgery (CAS) were recruited at the Pitié-Salpêtrière Hospital after approval of the study protocol by the Ethics Committee for Human Research of this hospital (Session of April 4^th^, 2007). The following patients were excluded: those undergoing coeloscopic surgery or surgery on the thoracic aorta, those with signs of pre-operative infection, undergoing chronic dialysis, under anti-inflammatory medication or antibiotic treatment before surgery, presenting an on-going or neoplastic hematologic pathology, or in an immunodepressed state. Finally, 20 AAS patients (17 males and 3 females; age 67.0 ± 2.9 years) and 20 CAS patients (13 males and 7 females; age 73.9 ± 2.8 years) were included in this study. There were no significant differences in age or proportion of gender between the two surgery groups. The two groups showed similar medical history (that is, hypertension, diabetes mellitus, angina pectoris, myocardial infarction, heart failure, coronary bypass, chronic obstructive pulmonary disease, renal failure). The protocol followed for preoperative medication and anesthesia was similar in both groups of patients. The only difference was that treatment with anti-platelet aggregation agents was discontinued five days before surgery for AAS patients, whereas it was continued until the day of surgery for CAS patients. The usual premedications were maintained except for converting enzyme inhibitors and angiotensin II antagonists, which were discontinued the day before surgery. All patients were premedicated with 5 mg of midazolam given orally one hour before surgery. During the operative period, all patients were anesthetized by target-controlled infusion of propofol, sufentanil, and cisatracurium. Antibioprophylaxis was performed using cefamandole. Depending on patient hemodynamics and hematocrit, fluid loading was performed using crystalloid infusion (lactated Ringer's solution or isotonic saline) and colloid infusion (hydroxyethylstach 130/0.4), associated with blood transfusion if necessary to maintain hemoglobin levels above 10 g/dl. Approximately 30 minutes before the end of surgery, all patients received paracetamol for postoperative analgesia, which was completed in the recovery room with intravenous morphine until pain relief was achieved. Healthy volunteers were recruited (ICaReB) in order to determine the main mediators responsible for the down-regulation of HLA-DR expression on CD14^HIGH ^and CD14^LOW ^monocyte subpopulations (n = 9, three males and six females; age 37 ± 5 years). Informed consent was obtained from each patient and volunteer.

### Blood sampling

Blood samples from patients were collected into sodium citrate vacuum tubes as follows: immediately before anesthesia induction (T_1_); before incision (T_2_), before vascular clamping (aortic clamping (AAS patients) or carotid artery clamping (CAS patients)) (T_3_), after blood reperfusion (T_4_) during the surgery, and on postoperative Days 1 (POD1) and 2 (POD2) after the surgery. Blood samples from some patients were collected on POD4 (CAS patients, n = 7) or POD7 (AAS patients, n = 10). Blood from healthy controls (12 ml/each) was collected into sodium citrate vacuum tubes.

### Flow cytometric analysis

Whole blood (100 μl) was immediately processed for double staining with 20 μl of fluorescein isothiocyanate (FITC)-anti-HLA-DR antibody (Beckman Coulter, Marseille, France) or 20 μl FITC-anti-CD16 antibody (Beckman Coulter) and 4 μl of phycoerythrin (PE)-anti-CD14 antibody (MY4-RD1, Beckman Coulter, Fullerton, CA, USA). For IL-10 receptor expression, 100 μl of whole blood was incubated with 10 μl of FITC-anti-CD14 antibody (MY4, Beckman Coulter), 10 μl of allophycocyanin (APC)-anti-CD16 antibody (Miltenyi Biotec, Bergisch Gladbach, Germany) and 20 μl of PE-anti-IL-10R (CD210) antibody (Biolegend, San Diego, CA, USA). As isotype controls, 2 μl of FITC-mouse IgG_1 _or IgG2b (Sigma-Aldrich, St Louis, MO, USA), 10 μl of PE-mouse IgG_2_a or IgG2b (Miltenyi Biotec) and/or 10 μl of APC-mouse IgM (Miltenyi Biotec) were used. After 20 minutes of incubation in the dark, 1 ml of lysis buffer (BD FACS™ lysing solution, BD Bioscience, Franklin Lakes, NJ, USA) was added to stained samples to lyse erythrocytes. After a further 10-minute incubation and centrifugation (300 g for five minutes, 4°C), the supernatant was removed and 300 μl of MACS buffer (DPBS with 2 mM EDTA and 0.5% fetal calf serum) was added to cells. The expression of surface markers was immediately measured by flow cytometry (FACScan, BD Bioscience). The settings of the flow cytometer were maintained constant during the whole study, which was performed with the same batch of antibodies for all patients, allowing a similar signal for the monocyte subsets throughout the investigation. The values were expressed as mean fluorescence intensity (MFI). Data analysis was performed using CellQuest software (BD Bioscience, Franklin Lakes, NJ, USA).

### Screening of mediators responsible for the down-regulation of HLA-DR expression on CD14^HIGH ^and CD14^LOW ^monocytes

Whole blood samples from healthy volunteers were incubated with each or a combination of the following molecules for 24 hours (37°C, 5% CO_2_): norepinephrine (MERCK, Lyon, France), acetylcholine (Sigma-Aldrich), vasoactive intestinal peptide (VIP) (Sigma-Aldrich), pituitary adenylate cyclase-activating polypeptide (PACAP) (Sigma-Aldrich), substance P and enkephalin (kind gifts of Dr Catherine Rougeot, Institut Pasteur), transforming growth factor-β (TGF-β) (R&D Systems, Abingdon, Oxfordshire, UK), tumor necrosis factor-α (TNF-α) (R&D systems), interleukin-10 (IL-10) (Genzyme, Saint Paul, MN, USA), prostaglandin E_2 _(PGE_2_) (Sigma-Aldrich), adrenocorticotropic hormone (ACHT) (Novartis, Rueil-Malmaison, France), glucocorticoid (hydrocortisone, HC) (Sigma-Aldrich), blocker of corticoid receptor (RU486; mifepristone, Sigma-Aldrich), and pathogen-associated molecular patterns (PAMPs) (Pam3CysSK4 (EMC microcollection, Tübingen, Germany), muramyl dipeptide (MDP; Sigma-Aldrich), *E. coli *lipopolysaccharide (LPS; Alexis, Enzo Life Sciences Inc., Farmingdale, NY, USA)). The cells were then stained and flow cytometry was performed following the procedure described above.

### Measurement of plasma cortisol and interleukin-10

Plasma levels of cortisol before anesthesia (T_1_) and at POD1 were measured using enzyme immunoassays (AbCys S.A., Paris, France). Plasma levels of IL-10 were measured by an enzyme-linked immunosorbent assay (ELISA) (DuoSet, R&D Systems). The assays were carried out according to the manufacturer's instructions. The resultant color reaction was read using a MRX ELISA microplate reader (Revelation, DYNEX, Magellan Science, Gaithersburg, PA, USA) at 450 nm.

### Incubation of blood from healthy volunteers with plasma from surgery patients

Whole blood from healthy volunteers was centrifuged and the plasma replaced with that from AAS or CAS patients collected after blood reperfusion (T_4_). The samples were incubated for 24 hours (37°C, 5% CO_2_). In some samples, RU486 (20 μM), a glucocorticoid receptor antagonist, was added simultaneously. The cells were stained and flow cytometry was then performed following the procedure described above to determine HLA-DR expression. The results, expressed as the mean of % change of HLA-DR expression, were compared to HLA-DR expression after 24 hours incubation at 37°C in the presence of autologous plasma.

### Quantitative real-time PCR for MARCH1 gene expression

Whole blood was subjected to Ficoll separation (MSL, Les Ullis, France) in order to isolate peripheral mononuclear cells (PBMCs). Monocytes were isolated from PBMCs using MACS CD14 magnetic beads (Miltenyi Biotec), and total RNA was extracted using the RNeasy miniprep kit (Qiagen, Valencia, CA, USA) following the manufacturer's protocol. cDNA was generated by reverse transcription as previously described [31]. Quantitative real-time polymerase chain reaction (qPCR) was performed on a Stratagene MX3005P^® ^using Brilliant^®^II SYBR^®^Green qPCR Master mix (Agilent Technologies, Massy, France), and 10 μM of each primer (custom synthesis by Sigma Oligo, St Louis, MO, USA). Primer sequences for MARCH1 are the following: hMARCH1 E1-258 F1 *TCCCAGGAGCCAGTCAAGGTT*, hMARCH1 E2-385 R1 *CAAAGCGCAGTGTCCCAGTG *[23]. The PCR consisted of 40 cycles at 94°C for 40 sec, 58°C for 30 sec and 72°C for 40 sec. The specificity of the SYBR green-amplified product was confirmed by dissociation curve analysis. Transcript levels for the MARCH1 gene were normalized against those of the housekeeping gene GAPDH [32].

### Statistical analysis

Levels of HLA-DR expression on the two CD14 positive monocyte subpopulations before, during and after surgery in each patient group were examined by repeated measure one-way analysis of variance (ANOVA) followed by least significant difference (LSD) post-hoc tests. General characteristics and other biological variables between the two patient groups or between non-modulated and modulated blood samples were tested by the Mann-Whitney U-test, the Wilcoxon signed-rank test or the Fischer's exact test depending on the data. The relationship between plasma levels of cortisol or IL-10 and the modification of HLA-DR expression on monocyte subpopulations was evaluated using Spearman's rho coefficient. *P*-values less than 0.05 were considered significant. All statistical analyses were performed using SPSS version 12.0 for Windows (Statistical Package for the Social Science, SPSS Ins., Chicago, IL, USA).

## Results

### Patients' characteristics

As shown in Table 1, the group of patients who underwent AAS was not statistically different from those who underwent CAS. In contrast, all parameters linked with this type of surgery indicate that AAS was more severe than CAS in terms of duration, length of clamping, blood loss, transfusion, and translocation of microbial products from the gut [33,34]. This difference in severity was illustrated by significantly higher levels of markers of inflammation (IL-6, C-Reactive Protein (CRP)) one day after surgery in AAS patients. In addition, post-operative complications were more frequent in AAS: nine AAS patients and three CAS had cardiac and/or pulmonary complications (*P *= 0.038). Infections occurred in some of the patients with post-operative infection without reaching statistical significance between AAS and CAS patients.

**Table 1:** Patients, surgical procedure and survey characteristics, and levels of IL-6, CRP and cortisol on postoperative Day 1 (POD1)

	AAS patients (n = 20)	CAS patients (n = 20)	*P*-value
Age (years)	67.0 ± 2.9	73.9 ± 2.8	ns
Male/female (n)	17/3	13/7	ns
Blood loss (mL)	1,000 (400-3,500)	100 (50-900)	< 10^-4^
Fluid infusion (mL)	4,500 (3,000-9,000)	1,500 (1,000-3,000)	< 10^-4^
Red blood cell transfusion	1 (0 to 6)	0 (0 to 2)	< 10^-4^
Fresh-frozen plasma	1 (0 to 4)	0	ns
Cell-saver	2 (0 to 8)	0	ns
Operation duration (hours)	2.5 (1.6 to 6.5)	1.2 (1 to 2.5)	< 10^-4^
Vascular clamping duration (minutes)	50.5 (14 to 90)	26 (12 to 45)	< 10^-4^
Post-surgical complications (cardiac and/or respiratory)	9	3	0.038
Infection	4	2	ns
IL-6 pg/ml (POD1)	142 ± 41	14 ± 3	0.004
CRP pg/ml (POD1)	132 ± 16	25 ± 4	< 0.001
Cortisol ng/ml (POD1)	229 ± 44	157 ± 26	0.07

Results are provided either as the mean ± SEM or as the median (range).

Continuous values were compared by the Mann-Whitney test and frequencies or percentages were tested by the χ^2 ^or Fisher exact test. *P *values are given for the comparison between AAS and CAS. ns: not significantly different.

### Monitoring of HLA-DR expression on CD14^HIGH ^and CD14^LOW ^monocyte subpopulations in patients before, during, and after surgery

**Figure 1:**
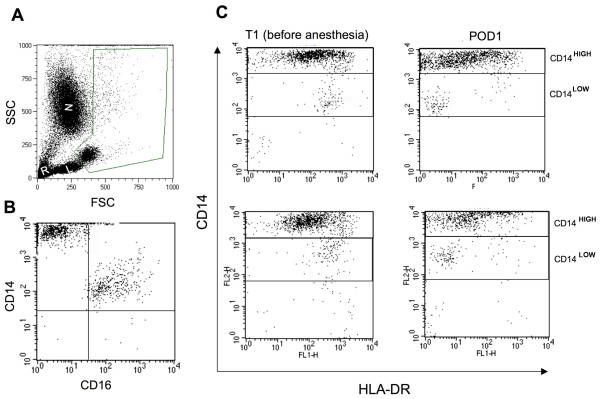
**Representative flow cytometry analysis of HLA-DR expression on CD14^HIGH ^and CD14^LOW ^monocyte subsets**. Representative flow cytometry analysis of HLA-DR expression on CD14^HIGH ^and CD14^LOW ^monocyte subsets during and after surgery. **(A) **Flow cytometric analysis was performed on whole blood samples after elimination of neutrophils (N), lymphocytes (L) and red cells (R) using the forward scatter (FSC) and side scatter (SSC) characteristics. **(B) **A representative dot-plot showing that CD14^HIGH ^monocytes were CD16^- ^and CD14^LOW ^monocytes were CD16^+ ^in a patient before surgery. **(C) **Monocytes were analyzed using an anti-CD14 antibody coupled with phycoerythrin (PE) and an anti-HLA-DR antibody coupled with fluorescein isothiocyanate (FITC). Representative flow cytometric analysis for HLA-DR expression on CD14^HIGH ^and CD14^LOW ^monocytes performed before anesthesia (T_1_) and on postoperative Day 1 (POD1) are shown for two abdominal aortic surgery (AAS) patients.

Monocytes were analyzed after exclusion of the other cells using side scatter (SSC) and forward scatter (FSC) parameters (Figure 1A). We also checked that CD14^HIGH ^monocytes were CD16^- ^and that CD14^LOW ^monocytes were CD16^+^, as previously reported [35] (Figure 1B). Similar patterns were obtained for patients before surgery and for healthy controls (data not shown). HLA-DR expression before surgery in AAS and CAS patients was not significantly different from that measured in healthy volunteers. HLA-DR MFI absolute values for AAS and CAS before anesthesia and healthy controls on CD14^HIGH ^monocytes were 282 ± 31, 285 ± 23 and 211 ± 37, respectively. HLA-DR MFI absolute values for AAS and CAS before anesthesia and healthy controls on CD14^LOW ^monocytes were 426 ± 34, 418 ± 31 and 452 ± 31, respectively. Figure 1C shows a representative flow cytometric analysis of HLA-DR expression on CD14^HIGH ^and CD14^LOW ^monocyte subpopulations monitored at T_1 _(before anesthesia) and POD1 for two AAS patients. It can be seen that HLA-DR expression on CD14^HIGH ^and CD14^LOW ^monocytes at POD1 was decreased as shown by a leftward shift as compared to levels at T_1_.

**Figure 2:**
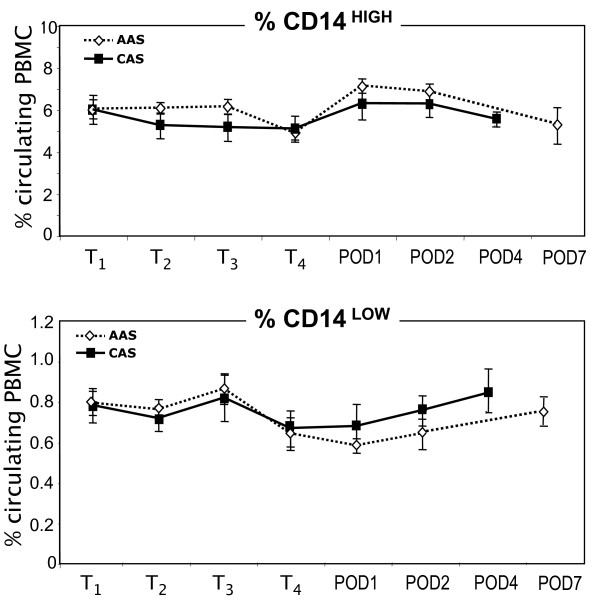
**Survey of CD14-positive subsets during and after surgery**. Percent of CD14^HIGH ^and CD14^LOW ^subsets was followed in abdominal aortic surgery (AAS, close symbol) and carotid surgery (CAS, open symbol) patients, at T_1 _(before anesthesia), T_2 _(before incision), T_3 _(before clamping), T_4 _(after reperfusion), and on post-operative day 1, 2, 4 and 7 (POD1, 2, 4, 7). The results are expressed as percent of CD14-positive cells among leukocytes.

At T_1_, the percentage of CD14^HIGH ^monocytes was of 6.02 ± 0.45 and 5.99 ± 0.69, and that of CD14^LOW ^was of 0.80 ± 0.06 and 0.78 ± 0.08 for AAS and CAS patients respectively. These values were similar to those obtained with healthy donors, and didn't vary significantly during the survey (Figure 2).

**Figure 3:**
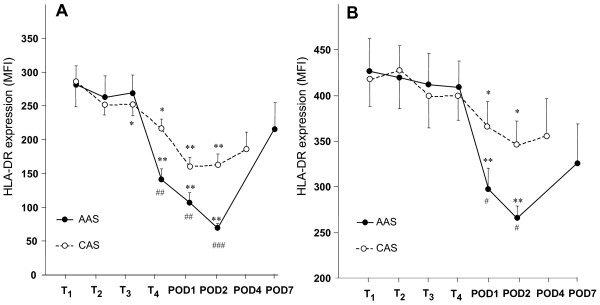
**Monitoring of HLA-DR expression on CD14^HIGH ^and CD14^LOW ^monocyte subsets during and after surgery**. Mean ± SEM corresponding to the mean fluorescence intensity (MFI) for HLA-DR expression on CD14^HIGH ^**(A) **and CD14^LOW ^**(B) **monocytes from patients undergoing abdominal aortic surgery (AAS, close symbol) or carotid surgery (CAS, open symbol) at T_1 _(before anesthesia), T_2 _(before incision), T_3 _(before clamping), T_4 _(after reperfusion), and on post-operative Days 1, 2, 4 and 7 (POD1, 2, 4, 7). The two curves in both panel A and B are significantly different (*P *= 0.001). * *P *< 0.05, ** *P *< 0.001 compared with the initial value (T1) for each monocyte subset. # *P *< 0.05, ## *P *< 0.01 and ### *P *< 0.001 when comparing the two groups of patients at one given time point.

Figure 3A shows that in AAS patients, HLA-DR expression on CD14^HIGH^cells was reduced after blood reperfusion (T_4_) (-49%) and continued to decrease by POD1 (-60%) and POD2 (-73%). In contrast, the expression of HLA-DR on CD14^LOW ^cells did not change at T_4_, and was only reduced at POD1 (-40%) and POD2 (-51%) (Figure 3B). The kinetics for the two subpopulations were significantly different (*P *< 0.01). On POD4 or POD7, HLA-DR expression on both subpopulations returned to close to the normal range as measured before surgery. AAS patients were compared with a second group of patients (CAS) for whom the inflammatory insult was less severe. For CAS patients, the kinetics of the reduction of HLA-DR expression on CD14^HIGH ^and CD14^LOW ^cells were not different of that of AAS patients, but the reduction was significantly less severe.

### Relationship between plasma levels of cortisol or IL-10, and the modulation of HLA-DR expression on CD14^HIGH ^and CD14^LOW ^monocytes

**Figure 4:**
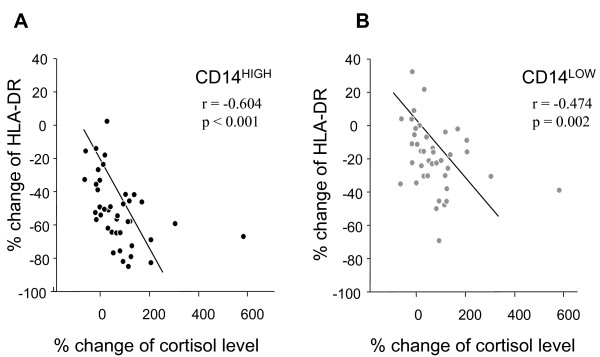
**Correlation between percent change in plasma cortisol levels and in HLA-DR expression on CD14^HIGH ^and CD14^LOW ^monocytes**. Percent change was calculated between the levels observed before anesthesia (T_1_) and on postoperative Day 1 (POD1). Analysis with Spearman's rho correlation coefficient was performed for all 40 patients (AAS and CAS). **(A) **CD14^HIGH ^monocytes; **(B) **CD14^LOW ^monocytes.

We measured plasma levels of cortisol and IL-10 in AAS and CAS patients in order to investigate their possible relationship with the modulation of HLA-DR expression on both CD14 monocyte subpopulations. Plasma cortisol levels before anesthesia (T_1_) were not significantly different among AAS patients (110.8 ± 8.7 ng/ml), CAS patients (104.8 ± 10.8 ng/ml) or healthy controls (95.5 ± 10.7 ng/ml). Both groups of patients showed a significant increase in plasma levels of cortisol at POD1 (Table 1), in agreement with previous studies, which reported peak level of cortisol one day after surgery [36,37]. A negative correlation between the percent change in the levels of plasma cortisol and the percent change in HLA-DR expression on CD14^HIGH ^and CD14^LOW ^monocytes was observed when comparing values obtained at T_1 _and POD1 (Figure 4).

Plasma levels of IL-10 were measured during the observational period in both groups of patients. The levels were almost undetectable until T_3 _in both groups. A peak of IL-10 in the AAS group occurred at T_4 _(mean ± SEM (standard error of the mean) = 30 ± 9 pg/ml; median = 15.0 pg/ml) and IL-10 could be still detected at POD1 (mean ± SEM = 23 ± 6 pg/ml, median = 17.5 pg/ml), whereas IL-10 was below the detection limit in most CAS patients. Thus, the relationship between changes in HLA-DR expression and IL-10 levels could only be analyzed in AAS patients. However, the absence of detectable IL-10 in CAS patients does not mean that there was no IL-10 produced, since detectable circulating cytokines only represent the tip of the iceberg [38]. A significantly negative correlation between IL-10 levels and the alteration of HLA-DR expression could be obtained for CD14^HIGH ^monocytes (r = -0.465, *P *= 0.039, when comparing T_1 _and T_4_, and r = -0.516, *P *= 0.030, when comparing T_1 _and POD1), but not for CD14^LOW ^monocytes.

### Deciphering the acting mediators associated with HLA-DR down-regulation on CD14^HIGH ^and CD14^LOW ^monocytes

**Figure 5:**
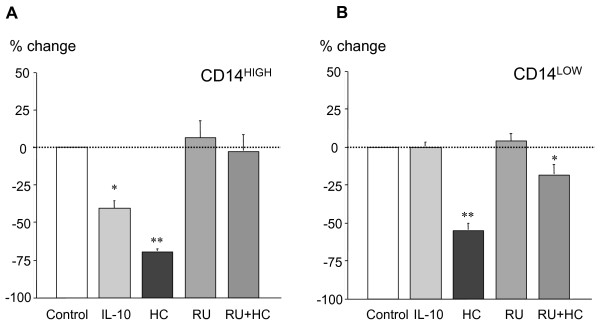
**Modulation of HLA-DR expression on CD14^HIGH ^and CD14^LOW ^monocytes by IL-10 or hydrocortisone**. Blood samples from healthy donors were incubated for 24 hours without or with IL-10 (10 ng/ml), hydrocortisone (HC, 100 μM), and/or RU486 (20 μM), an antagonist of the glucocorticoid receptor. For both subpopulations, the results are expressed as percent change of mean fluorescence intensity for HLA-DR measured in untreated cells (control). The results are the mean ± SEM of five independent experiments with different donors. * *P *< 0.05, ** *P *< 0.01 compared with control by Mann-Whitney U-test.

**Figure 6:**
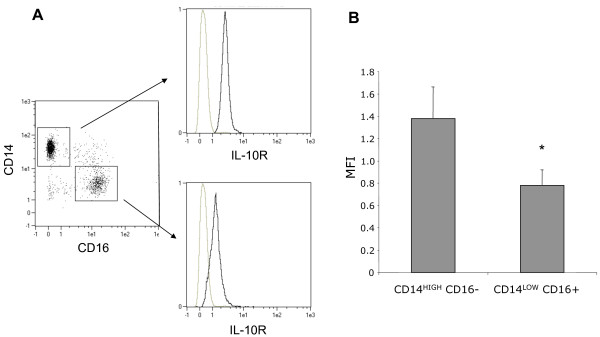
**Expression of the receptor for IL-10 (IL-10R) on CD14^HIGH ^and CD14^LOW ^monocytes**. Flow cytometric analysis was performed on whole blood from healthy volunteers after elimination of polymorphonuclear cells, red cells and debris, using the forward scatter (FSC) and side scatter (SSC) characteristics. **(A) **CD14^HIGH^CD16^- ^and CD14^LOW^CD16^+ ^monocytes were analyzed for the expression of IL-10R. **(B) **The mean fluorescence intensity (MFI) for each subset is shown. The results are the mean ± SEM of seven independent experiments with different donors (* *P *< 0.05 using the Wilcoxon signed-rank test).

In order to identify the mechanism of HLA-DR down-regulation on CD14^HIGH ^and CD-14^LOW ^monocytes, we tested various mediators produced during stress that are known to interfere with the immune response. Blood from healthy volunteers was incubated with each molecule or a combination of the molecules for 24 hours, and HLA-DR expression on both CD14^HIGH ^and CD14^LOW ^monocytes was analyzed by flow cytometry. IL-10 down-regulated HLA-DR expression on CD14^HIGH ^monocytes, but not on CD14^LOW ^cells (Figure 5). Hydrocortisone (HC) down-regulated HLA-DR expression on both monocyte subpopulations, and the effect of HC was inhibited by the glucocorticoid receptor antagonist RU486 (Figure 5). As the effect of IL-10 was not the same on the two monocyte subsets, we investigated whether this could be linked to a different expression of the receptor for IL-10 (IL-10R). The expression of the IL-10R was analyzed by flow cytometry on monocytes from healthy volunteers. As shown in Figure 6, its expression was significantly higher on the CD14^HIGH ^CD16^-^, the population that was sensitive to IL-10 effects *in vitro*.

**Figure 7:**
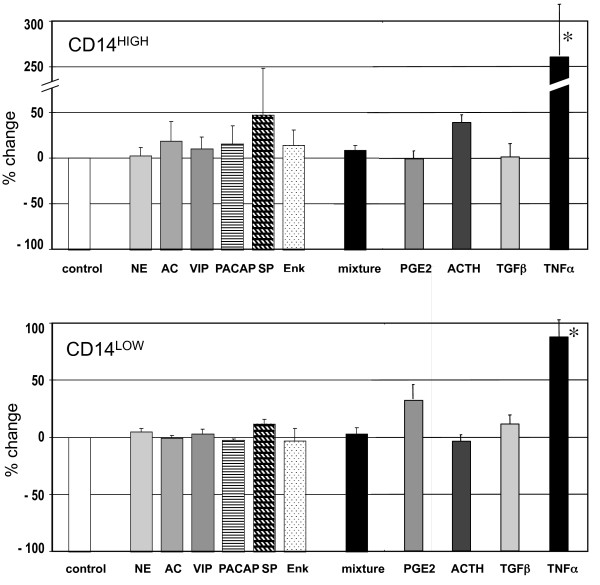
**Modulation of HLA-DR expression on CD14^HIGH ^and CD14^LOW ^monocytes by different neuromediators, cytokines, PGE_2 _and ACTH**. Blood samples from healthy donors were incubated for 24 hours without or with norepinephrine (NE, 10 nM), acetylcholine (AC, 10 μM), vasoactive intestinal peptide (VIP, 10 nM), pituitary adenylate cyclase-activating polypeptide (PACAP, 10 nM), substance P (SP, 1 μM), enkephalin (Enk, 1 μM), a mixture of all neuromediators, prostaglandin E_2 _(PGE_2_, 1 μM), adrenocorticotropin hormone (ACTH, 10 nM), transforming growth factor-β (TGF-β, 10 ng/ml), or tumor necrosis factor-α (TNF-α, 10 ng/ml). For both subpopulations, the results are expressed as the percent change of mean fluorescence intensity compared to HLA-DR measured in control cells. The results are the mean ± SEM of five independent experiments with different donors (* *P *< 0.05).

Neurotransmitters are among the other mediators associated with stressful situations. However, none of the tested neuromediators (norepinephrine, acetylcholine, vasoactive intestinal peptide, pituitary adenylate cyclase-activating polypeptide, substance P, and enkephalin) alone or together had any effect on HLA-DR expression (Figure 6). Similarly, prostaglandin E2, adrenocorticotropin hormone or TGFβ had no effect. In contrast, TNFα increased the expression of HLA-DR, particularly on CD14^HIGH ^cells (CD14^HIGH^: + 263%; CD14^LOW^: + 87%) (Figure 7).

**Figure 8:**
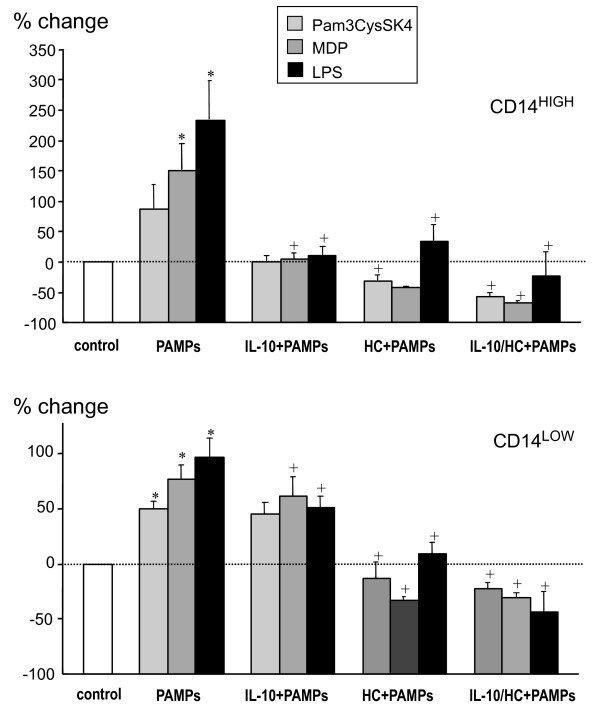
**Modulation of HLA-DR expression on CD14^HIGH ^and CD14^LOW ^monocytes**. Modulation of HLA-DR expression on CD14^HIGH ^and CD14^LOW ^monocytes by different PAMPs in the absence or presence of IL-10 and/or hydrocortisone. Blood samples from healthy donors were incubated for 24 hours without or with Pam3CysSK4 (100 ng/ml), muramyl dipeptide (MDP, 100 nM) or *Escherichia coli *LPS (100 ng/ml) in the absence or presence of IL-10 (10 ng/ml) and/or hydrocortisone (HC, 100 μM). For both subpopulations, the results are expressed as the percent change of mean fluorescence intensity compared to HLA-DR measured in untreated cells (control). The results are the mean ± SEM of four independent experiments with different donors. *P < 0.05, comparison between control and PAMPs --induced modulation. +*P *< 0.05, comparison between PAMPs alone and PAMPs + IL-10 and/or HC.

We previously showed that translocation of microbial products occurs in AAS patients [33,34]. Thus, we studied the capacity of several microbial products, including agonists of TLR2, TLR4 or NOD2 (Pam3CysSK4, LPS and MDP, respectively), to modulate the expression of HLA-DR on monocytes. As shown in Figure 8, HLA-DR expression on both CD14^HIGH ^and CD14^LOW ^monocytes was up-regulated by these pathogen-associated molecular patterns (PAMPs), particularly on CD14^HIGH ^cells. We then investigated the capacity of IL-10 or hydrocortisone to counteract the effects of these PAMPs. As compared to IL-10, hydrocortisone had a greater capacity to reduce the up-regulation of HLA-DR induced by PAMPs, especially on CD14^HIGH ^monocytes. When exposed to both IL-10 and HC, the levels were further reduced.

### Incubation of blood from healthy volunteers with plasma from AAS patients

We then investigated whether the plasma of AAS patients could suppress the expression of HLA-DR on CD14^HIGH ^and CD14^LOW ^monocyte subpopulations of healthy donors. Whole blood from healthy volunteers was incubated for 24 hours at 37°C, after replacing their plasma with that from AAS patients (n = 13) sampled at POD1. Decreased expression of HLA-DR was obtained for 69% of the plasma samples (9 out of 13 patients), with a 42 ± 9% decrease for CD14^HIGH ^and a 11 ± 8% decrease for CD14^LOW ^monocytes. In addition, for four AAS plasma, which led to a 37.3 ± 10.4% decrease of HLA-DR expression onto CD14^HIGH^monocytes from healthy controls, the co-incubation with RU486, a glucocorticoid receptor antagonist, fully abolished the inhibitory effect of the active AAS plasma (data not shown).

### Expression of MARCH1 in monocytes of healthy controls in the presence of hydrocortisone, and in monocytes of AAS patients after surgery

**Figure 9:**
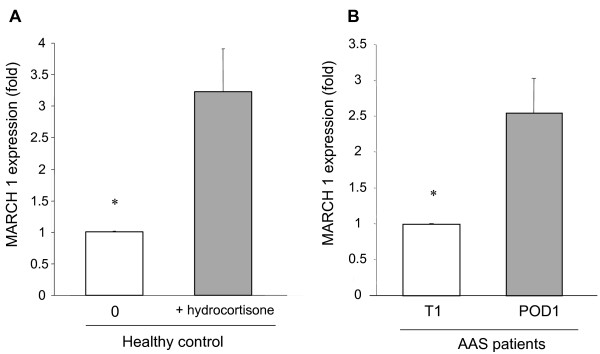
**Increased expression of MARCH1 in monocytes after treatment**. Increased expression of MARCH1 in monocytes after treatment with hydrocortisone *in vitro *or after abdominal aortic surgery. **(A) **MARCH1 mRNA expression in monocytes from healthy donors after a 24-hour incubation at 37°C in whole blood in the absence or presence of hydrocortisone (100 μM). MARCH1 expression was analyzed by qPCR and normalized as compared to GAPDH. The results are expressed as fold increase compared to untreated whole blood, and represent the mean ± SEM of six different donors. **(B) **MARCH1 mRNA expression in monocytes from AAS patients before anesthesia (T_1_) and one day post surgery (POD1). MARCH1 expression was analyzed by qPCR and normalized against that of GAPDH. The results are expressed as fold increase compared to the expression observed before surgery on T_1_, and represent the mean ± SEM of seven different patients. * *P *< 0.05 using the Wilcoxon signed-rank test.

Because of the known role of MARCH1 on HLA-DR trafficking [24,25], we investigated whether MARCH1 was modulated by glucocorticoids. As shown in Figure 9A, incubation of whole blood from healthy donors with hydrocortisone led to a three-fold increase in MARCH1 gene expression in their monocytes after 24 hours of incubation. Similarly, in AAS patients MARCH1 gene expression was increased at POD1 as compared to T_1 _(Figure 9B).

## Discussion

CD14 plays a key role in the endotoxin receptor complex and is expressed on both circulating monocytes and neutrophils. Monocytes express higher levels of CD14 than neutrophils. However, there is a CD14^LOW^CD16^+ ^subpopulation among circulating monocytes that accounts for about 10% of all blood monocytes [27]. This subpopulation resembles tissue macrophages, is increased in many inflammatory disorders [39] and is the major source of TNF [40], whereas the IL-10 transcript is absent or present at low levels [41].

All monocytes express the HLA-DR molecule, and levels are greatly decreased during stressful situations (for example, trauma, severe surgery, sepsis, pancreatitis, burn, hemorrhagic shock, and transplantation). The degree and the duration of HLA-DR reduction on monocytes are associated with the occurrence of nosocomial infections and outcome [8-18]. In this study, we found that the recovery of normal HLA-DR expression for AAS patients occurred faster than in other patients undergoing a similar surgery but with more severe stress, such as ruptured abdominal aortic aneurism [12]. The present study was aimed to analyze HLA-DR expression on monocyte subpopulations. We show for the first time that the expression of HLA-DR on the two monocyte subpopulations was not similarly down-regulated after a stressful situation such as an abdominal aortic surgery. AAS was chosen because blood samples could be harvested at different time points: before anesthesia (allowing us to have the initial values before the insult), during surgery (before clamping, and after reperfusion), and in the days following surgery in order to precisely analyze the kinetics of HLA-DR dowregulation. Furthermore, this procedure can be considered as a severe surgery associated with blood loss and translocation of PAMPs from the gut [33,34] that contributes to further enhancement of the post-operative inflammatory response. Indeed, CAS, a less severe surgery, was associated with a lower down-regulation of HLA-DR and less frequent post-operative complications. In AAS, decreased HLA-DR expression already occurred during surgery for the CD14^HIGH ^cells, earlier than that observed for the CD14^LOW ^population and with a more pronounced effect. Similar results were obtained with CAS patients, but to a lesser extent. Previous analysis performed on CD14^HIGH ^bright and CD14^LOW ^monocytes in patients undergoing cardiac surgery with cardiopulmonary bypass, or following low to intermediate risk surgery failed to detect a differential downregulation of HLA-DR on monocytes subsets [42,43]. This discrepancy with our study is most probably due to the fact that the surveys were not performed during surgery. Of course, our findings may relate more to changes in observed cell populations rather than to changes in HLA-DR expression by the individual cells since it were not the same cells that were analyzed at different time points.

The differential modulation of HLA-DR on monocyte subpopulations led us to consider that exogenous signals leading to this down-regulation could be different for each subset. We found increased levels of cortisol, and to a lesser extent of IL-10, after vascular surgery. The increase in cortisol levels observed in patients undergoing vascular surgery (both AAS and CAS) was negatively correlated with HLA-DR expression on both CD14^HIGH ^and CD14^LOW ^monocytes. In contrast, the correlation between levels of IL-10 and altered HLA-DR expression was only found for CD14^HIGH ^monocytes. Accordingly, we tested the capacity of IL-10 and glucocorticoids to down-regulate the expression of HLA-DR on either sub-population from healthy controls. While it was already known that both mediators down-regulated the expression of HLA-DR on monocytes [21,44-46], their specific effects on monocyte subpopulation was not investigated. In agreement with our *in vivo *observations, we showed that hydrocortisone was able to down-regulate HLA-DR expression on both monocyte subpopulations, whereas IL-10 only acted on CD14^HIGH ^monocytes. This later subpopulation showed a significantly higher expression of the IL-10R than the CD14^LOW^, which might explain the difference in sensitivity to this cytokine *in vitro*. These results also concur with the correlation found between HLA-DR expression on CD14^HIGH ^monocytes and IL-10 levels in AAS patients.

Plasma from AAS patients contains not only IL-10 and cortisol, but also other molecules that can differentially modulate the expression of HLA-DR, including cytokines (TNFα, TGFβ), translocated PAMPs, neuromediators, mediators of inflammation (PGE2) and stress (ACTH). None of the tested neuromediators, despite their known effects on immune cells [47-51] affected the expression of HLA-DR on monocyte subpopulations. In contrast, we showed that PAMPs such as LPS, Pam3CysSK4 and MDP were able to up-regulate the expression of HLA-DR on both monocyte subsets. Hydrocortisone and, to a lesser extent, IL-10 prevented the enhancement of HLA-DR expression by TLR2, TLR4 and NOD2 ligands. An inhibitory effect, similar to that of hydrocortisone was also observed with the plasma of many, but not all, AAS patients. One explanation might be that their plasma contains a complex mixture of enhancing and inhibitory agents, the ratio of which may change with time, and not always result in a reduction of the expression of HLA-DR. This concept is illustrated by *in vitro *enhancement of HLA-DR expression on monocytes by LPS when in contrast, a reduced expression was observed on monocytes isolated from human volunteers injected with LPS [52].

Fumeaux and Pugin [22] showed that IL-10 induces internalization of surface HLA-DR molecules, and Le Tulzo et al. [21] reported that glucocorticoids inhibit the synthesis of mRNA coding for HLA-DR. In septic patients, globally decreased expression of genes involved in HLA-DR surface expression has been reported [53]. In agreement with these reports, we observed a global decrease in HLA-DR expression as determined by flow cytometry after treatment with hydrocortisone, both on the surface, and intracellularly after cell permeabilization (data not shown). Finally, in order to gain insight into the mechanism of HLA-DR down-regulation by glucocorticoids, we analyzed the expression of MARCH1. This molecule is known to increase the intra-cellular sequestration of HLA-DR [23] as well as its ubiquitination [25], and to decrease its half-life [24]. In the present study, we showed for the first time the capacity of glucocorticoids to up-regulate the expression of MARCH1 mRNA in monocytes from healthy controls. Most importantly, we observed an up-regulation of MARCH1 mRNA *in vivo *in monocytes from AAS patients one day after surgery.

## Conclusions

We report for the first time that following a stressful situation, the down-regulation of HLA-DR expression on the two monocyte subsets, namely CD14^HIGH ^(*classical*) and CD14^LOW ^(*inflammatory*), neither occurs simultaneously nor in response to the same mediators. The HLA-DR downregulation on the CD14^LOW ^subset, which is increased during sepsis [29], was transient and less severe. Furthermore, our data suggest that MARCH1 up-regulation by glucocorticoids might be a key element leading to reduced expression of HLA-DR on both CD14^HIGH ^and CD14^LOW ^monocytes. In contrast, IL-10-induced HLA-DR down-regulation only occurs among CD14^LOW ^CD16^+ ^monocytes.

## Key messages

• Down-regulation of HLA-DR on monocytes during systemic inflammation does not occur with similar kinetics among CD14^HIGH ^and CD14^LOW ^subsets.

• Among mediators involved in the down-regulation of HLA-DR on monocytes, glucocorticoids act on both CD14^HIGH ^and CD14^LOW ^subsets, whereas IL-10 is only active on CD14^HIGH ^CD16^NEG ^monocytes, a subset that expresses higher levels of IL-10 receptors.

• Monocytes are exposed to concomitant signals that act in opposite directions, either up-regulating or down-regulating HLA-DR expression, and glucorticoids are the most efficient mediators to counteract the enhancing effects of microbial products.

• mRNA coding for MARCH1, a negative regulator of MHC class II, is up-regulated in patients' monocytes, and *in vitro *in monocytes of healthy controls upon exposure to glucocorticoids.

## Abbreviations

AAS: abdominal aortic surgery; AC: acetylcholine; ACTH: adrenocorticotropin hormone; APC: allophycocyanin; CARS: compensatory anti-inflammatory response syndrome; CAS: carotid artery surgery; Enk: enkephalin; FITC: fluorescein isothiocyanate; FSC: forward scatter; HC: hydrocortisone; HLA: human leukocyte antigen; HLA-DR: human leukocyte antigen class II; IL-: interleukin; IL-10R: IL-10 receptor; LPS: lipopolysaccharide; LSD: least significant difference; MARCH1: membrane-associated RING-CH-1 protein; MFI: mean fluorescence intensity; MDP: muramyldipeptide; NE: norepinephrine; PACAP: pituitary adenylate cyclase-activating polypeptide; PAMPs: pathogen-associated molecular patterns; Pam3CysSK4: tripalmitoylated lipopeptide (including one Cystein, one Serine, four Lysine); PBMC: peripheral blood mononuclear cells; PE: phycoerythrin; PGE_2_: prostaglandin E_2_; POD: postoperative days; qPCR: quantitative real-time polymerase chain reaction; SIRS: systemic inflammatory response syndrome; SP: substance P; TGF-β: transforming growth factor-β; TNF-α: tumor necrosis factor-α; VIP: vasoactive intestinal peptide.

## Competing interests

The authors declare that they have no competing interests.

## Authors' contributions

OYK analyzed the raw data, performed statistical analysis, and drafted and contributed to the writing of the paper. AM, MB and PC included patients, collected the clinical information, and approved the manuscript. JMC designed the study, analyzed the raw data and contributed to the writing of the paper. MAC designed the study, performed the experiments, analyzed the raw data, and drafted and contributed to the writing of the paper
